# MicroRNA retrocopies generated via L1-mediated retrotransposition in placental mammals help to reveal how their parental genes were transcribed

**DOI:** 10.1038/s41598-020-77381-8

**Published:** 2020-11-26

**Authors:** Cheng-Tsung Pan, Yeong-Shin Lin

**Affiliations:** 1 Institute of Bioinformatics and Systems Biology, National Chiao Tung University, Hsinchu, 300 Taiwan; 2 Department of Biological Science and Technology, College of Biological Science and Technology, National Chiao Tung University, Hsinchu, 300 Taiwan; 3 Center For Intelligent Drug Systems and Smart Bio-devices (IDS2B), National Chiao Tung University, Hsinchu, 300 Taiwan

**Keywords:** Non-coding RNAs, Gene duplication, Comparative genomics

## Abstract

In mammalian genomes, most retrocopies emerged via the L1 retrotransposition machinery. The hallmarks of an L1-mediated retrocopy, i.e., the intronlessness, the presence of a 3′ poly-A tail, and the TSDs at both ends, were frequently used to identify retrotransposition events. However, most previous studies only focused on protein-coding genes as their possible parental sources and thus only a few retrocopies derived from non-coding genes were reported. Remarkably, none of them was from microRNAs. Here in this study, we found several retrocopies generated from the mir-302–367 cluster gene (*MIR302CHG*), and identified a novel alternatively spliced exon encoding mir-302a. The other recognized microRNA retrotransposition events are primate-specific with mir-373 and mir-498 as their parental genes. The 3′ poly-A tracts of these two retrocopy groups were directly attached to the end of the microRNA precursor homologous regions, which suggests that their parental transcripts might alternatively terminate at the end of mir-373 and mir-498. All the three parental microRNAs are highly expressed in specific tissues with elevated retrotransposon activity, such as the embryonic stem cells and the placenta. This might be the reason that our first microRNA retrocopy findings were derived from these three microRNA genes.

## Introduction

The term ‘retrocopies’ refers to both functional retroposed genes (retrogenes) and processed pseudogenes, and in mammals, which are mainly generated via the retrotransposition (or retroduplication) machinery mediated by long interspersed nuclear element-1 (L1 or LINE-1) activity^1^. L1 elements are a group of autonomous non-LTR retrotransposons abundant in mammalian genomes and currently the only autonomously active one in humans^2,3^. In addition to retrotransposing their own L1 transcripts, the enzymes encoded by L1 elements could also retrotranspose non-L1 transcripts^2,4^. The retrotransposition process starts from the reverse transcription of a parental gene’s mRNA, which generates a complementary DNA (cDNA) that could subsequently be inserted into a new genomic location as a retrocopy^5,6^. During the course of retrotransposition, two copies of a short fragment, which sandwich the inserted cDNA sequence, are generated and known as flanking direct repeats (FDRs) or target site duplications (TSDs). The TSDs accompanied by the intronless insertions and the presence of poly-A tracts on the 3′ tail of insertions are the three well-studied hallmarks of L1-mediated retrotransposition^1,7,8^.

In recent years, due to the development of cutting-edge genome sequencing, numerous genome-wide retrotransposition analyses have been published^9,10^. However, most of these studies focused on the cases of retrocopies with protein-coding genes as their parental genes and therefore relied on the identification of exon-exon junctions or alternative splice sites as the evidence of processed pseudogenes. Although utilizing this strategy may help to find most retrocopies duplicated from protein-coding genes, the others derived from non-coding genes without splicing might be omitted. Thirty years ago, Brosius first speculated that the L1-mediated retrotransposition mechanism for protein-coding genes should also be applied to non-coding genes with or without splicing patterns^7^. In the following years, more studies had mentioned the possibility that novel non-coding genes could be duplicated through retrotransposition^11,12^. As expected, two later studies had identified L1-mediated retrotransposition derived from non-coding genes, including rRNAs, snRNAs, snoRNAs, tRNAs, 7SK RNAs, and 7SL RNAs in humans and chimpanzees^13^, and U6 snRNA in mammals^14^. Be that as it may, microRNAs were not included in their analyses. Another genome-wide study had investigated a broad set of expressed coding and non-coding retrocopies in primate genomes^15^; however, no microRNA retrocopies were reported either.

MicroRNAs are a kind of non-coding small RNAs (~ 22 nt) that regulate gene expression by either mRNA degradation or translational suppression^16,17^. In most cases, mammalian microRNA genes are harbored within introns of protein-coding genes or other non-coding RNAs that are transcribed by canonical RNA polymerase II (Pol-II) machinery^18–21^. These intronic microRNAs are co-expressed as byproducts of the mRNA splicing process^19,22^. In contrast, intergenic microRNA genes have their own promoters and are independently transcribed.

Although microRNA genes are widely dispersed in the genome, their distributions are not entirely random^23,24^; a portion of microRNAs tends to form clusters and be co-expressed together^25–27^. These microRNAs within a cluster are expressed via a polycistronic transcript and transcribed as a single unit^21,28^. Most of these microRNA clusters are lineage-specific with highly conserved members^29^, such as the vertebrate-specific mir-302–367 cluster^30^. The microRNA biogenesis machinery begins with the Drosha-mediated cleavage of stem-loop structures embedded in long primary transcripts (pri-miRNA), and accordingly generates the hairpin-structured microRNA precursors (pre-miRNA). The precursors are further processed by Dicer-mediated cleavage into single-stranded microRNA matures, which are the functional forms in length of ~ 22 nt^31–33^.

**Figure 1:**
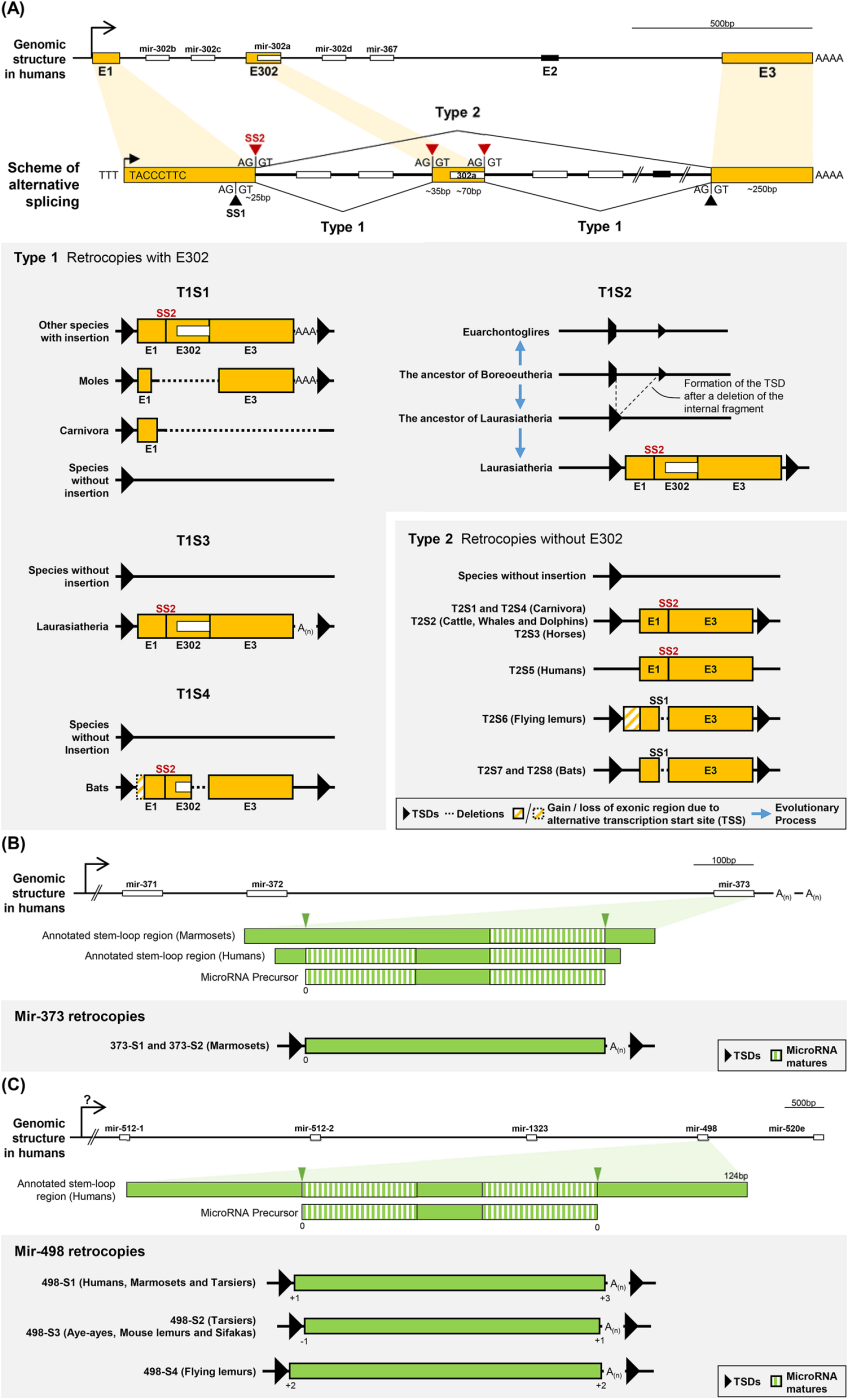
The genomic structures of the identified retrocopies and their parental genes. (**A**) Two types (T1 and T2) of alternative splicing for *MIR302CHG*, i.e., with or without the mir-302a-encoding exon E302, and all the derived retrocopies at different insertion sites (S1 ~ S4 for T1 and S1 ~ S8 for T2) are displayed. The yellow and white rectangles represent the exons and microRNA stem-loop regions, respectively. The black rectangle is the second exon E2 currently annotated in the human genome; however, this exon does not exist in all our investigated retrocopies. The red triangles denoted in the scheme of alternative splicing are splice sites newly identified in this study, while the black ones have been previously annotated in the human genome as well. The alternative usage of splice sites SS1 and SS2 determine the length of exon E1. (**B**) Mir-373 retrocopies and (**C**) mir-498 retrocopies were derived from the entire microRNA precursors. The green inverted triangles indicate the Drosha cleavage sites inferred from matures in miRBase. The 5′ end cleavage site of marmoset mir-373 precursor is inferred from the mature of human mir-373.

While we studied the evolution of mir-302–367 cluster (unpublished data), we occasionally found several unannotated paralogs of mir-302a precursor (a member of this cluster) in most mammals. Based on the identified hallmarks, we speculated that these paralogs might be duplicated from mir-302a via the L1-mediated retrotransposition during mammalian evolution. This mir-302–367 cluster is composed of four mir-302 paralogs (mir-302b, mir-302c, mir-302a, and mir-302d) and mir-367, which are vertebrate-specific and predominantly expressed in embryonic stem cells (ESCs)^30,34–36^, implying their crucial function in vertebrate development. The host gene of mir-302–367 cluster, *MIR302CHG*, was annotated to have three alternative transcripts in humans (NCBI accession numbers NR_146092 ~ NR_146094). The gene structure and the promoter of NR_146093 have been well-characterized^35^, which contains three exons labeled as E1, E2, and E3 in this study (Fig. 1). In contrast, NR_146094 has only E1 and E3. The mir-302–367 cluster locates within the intron between E1 and E2 and is likely expressed as an intronic byproduct of the primary transcript generated by the canonical Pol-II transcription system^35^. The 3′ poly-A tail provides the canonical Pol-II transcript an opportunity to serve as a source gene for L1-mediated retrotransposition.

The other primate-specific retrotransposition cases found in this study were derived from mir-373 and mir-498 precursors. Mir-373 is the third microRNA member of the mammalian-specific mir-371–373 cluster. Similarly, mir-498 is the third microRNA member harbored in C19MC (chromosome 19 microRNA cluster, located ~ 25 kb upstream of the mir-371–373 cluster in humans) with mir-512 and mir-1323 as the first and second members, respectively. C19MC is generally regarded as a primate-specific microRNA cluster that contains many conserved and tandemly repeated exons^22^. As the largest microRNA cluster in humans, it harbors 46 microRNAs within ~ 100 kb genomic region^29^. The tissue-specific expression pattern of both microRNA clusters (the mir-371–373 cluster is predominantly expressed in embryonic stem cells^34,36,37^ while C19MC is mainly expressed in the placenta^38–40^ and primary human trophoblast cells^41^) suggests their essential roles involved in the developmental stages. However, in contrast to the well-characterized mir-371–373 cluster that has a putative conserved promoter validated in mice^42^, the transcriptional mechanism of individual C19MC members is still unclear. One possible scenario is that each microRNA member could be transcribed separately from the promoter provided by its proximal upstream Alu element (a kind of primate-specific SINEs, short interspersed nuclear elements) via the polymerase III machinery^43^. Alternatively, microRNA members might be expressed as intronic byproducts via an alternative splicing process with some of these conserved and tandemly repeated C19MC exons involved^22^. This mechanism ought to adopt polymerase II machinery and might be regulated by a distal imprinted CpG island^38^. The discovery of retrocopies that emerged from mir-498 in this study may provide insight to address this debate.

## Results

### The retrocopies derived from the *MIR302CHG* gene in Placentalia

To comprehensively collect mir-302a related sequences, we used an iterative search strategy to scan genomes from 40 placental mammals, four marsupials and the platypus (see Materials and Methods and Table S1). Incorporating syntenic information, we successfully identified multiple retrocopies that emerged from independent insertion events (Fig. 1 and Table S2). The typical hallmarks of L1-mediated retrotransposition, including the TSDs at both ends and the poly-A tract on 3′ end of inserted sequences, were recognized in all these retrocopies (Fig. S1). By aligning these retrocopies with their parental genes *MIR302CHG*, we identified three exons (E1, E302, and E3) constituting retrocopies either with or without the middle one (E302) (Fig. 1). The above findings suggest that, similar to protein-coding genes, these retrocopies were probably originated from processed, polyadenylated long non-coding transcripts via the L1-mediated retrotransposition mechanism.

The E1 and E3 exons were named according to the currently annotated human *MIR302CHG* gene (NR_146093). In contrast, E302 is a novel exon identified in this study and contains the region encoding mir-302a precursor in length of ~ 70 bp. In most investigated species, the E302 region of their *MIR302CHG* genes is sandwiched by the canonical GT-AG introns (Figs. 1 and S2). This finding further supports its exon status. We next examined next-generation sequencing (NGS) reads from human embryonic stem cells and found that some reads indeed span the junction between E1 and E302, and the junction between E302 and E3 (Fig. S3). Although these reads are rare and not the primary transcripts, this finding strongly supports the expression of the E302-included transcript in humans.

The E1 exon of retrocopies varies in length resulted from two different 3′ splice sites, SS1 and SS2. The site SS1 is currently annotated in NCBI for E1 of human *MIR302CHG* gene. However, we only found one flying lemur (F_Lemur)-specific and two bat-specific retrocopies generated from the transcripts processed by SS1. In contrast, SS2 is the site inferred from the rest of the retrocopies identified in this study (including the retrocopies found in humans). The E1 exon processed by SS2 is longer in the 3′ end than which processed by SS1 (Figs. 1 and S4).

**Figure 2:**
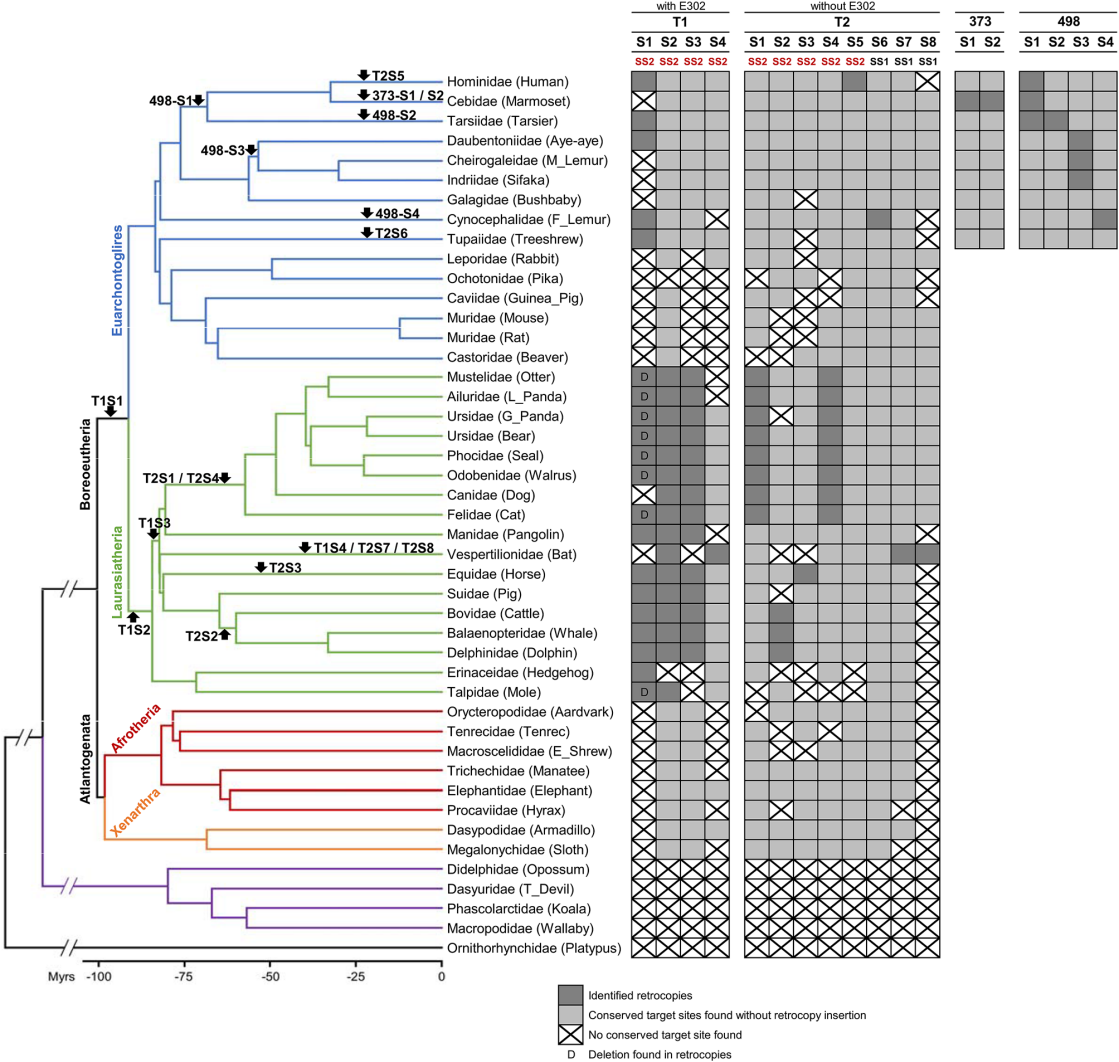
The presence or absence of the identified retrocopies for each retrotransposition event among various mammalian species. Eighteen independent retrotransposition events including two types of MIR302CHG retrocopies (T1 and T2), the mir-373 retrocopies (373), and the mir-498 retrocopies (498) with their unique insertion sites (four sites for T1, eight sites for T2, two sites for 373, and four sites for 498) are represented. The two alternative splicing sites, SS1 or SS2, used for each MIR302CHG retrotransposition event are also specified. The occurrence of each retrotransposition event is indicated by the corresponding arrow (only the relative position but not the precise dating) on the phylogenetic tree, which was adopted from Churakov et al.^54^ and Meredith et al.^55^. The retrocopies derived from mir-373 and mir-498 are all primate-specific, and thus their genomic analyses were conducted only in primates.

We further classified these MIR302CHG retrocopies into two types based on their alternative splice forms: with (type 1, T1) or without (type 2, T2, similar to the transcript NR_146094) exon E302. For each retrotransposition event, the presence or absence of certain retrocopies among species and the possible time period of occurrence are shown in Fig. 2. The inserted sequences, identified TSDs and the flanking sequences of these retrocopies were aligned and represented in the Supplementary MSA files.

### The MIR302CHG retrocopies encoding mir-302a homologs

Four independent T1 retrotransposition events were identified based on syntenic evidence. The four insertion sites (named from S1 to S4) include an intronic insertion within *IQCK* gene (T1S1), an intergenic insertion between *DIRC2* and *SEMA5B* genes (T1S2), an insertion in the opposite strand of the intron of *IDH2* gene (T1S3) and an intronic insertion of *CCDC150* gene (T1S4). Although we found a potential mammalian initiator embedded in the retroduplicated exon E1 and potential TATA-box motifs in the upstream regions of T1S1 and T1S2 retrocopies (Fig. S5), and the mir-302a homologous regions of some T1 retrocopies were inspected to have well-folded hairpin structures (Fig. S6), we found no evidence for their expression by analyzing four selected NGS microRNA expression datasets (miR-Seq) from NCBI Sequence Read Archive (SRA) database (Table S3). We further compared the mir-302a homologous regions between T1 retrocopies and their parental genes to investigate how conserved these sequences are. The results indicate that they are considerably diverged and could not reject the null hypothesis that the substitutions were neutrally accumulated (Table S4). It is likely that all these T1 retrocopies have turned into pseudogenes.

The T1S1 insertions exist in most species of Laurasiatheria (all species except dogs and bats) and some species of Euarchonta (humans, tarsiers, aye-ayes, flying lemurs and treeshrews), but not in the Glires clade. This extensive presence suggests T1S1 as the earliest event that emerged in the common ancestor of Laurasiatheria and Euarchontoglires lineages (Fig. 2). The homologous flanking regions of this insertion site were not identified in Glires, probably due to a complete deletion of this genomic area in their ancestor. Similarly, a particular region including partial E1, entire E302 and partial E3 was deleted in the retrocopy of moles (Fig. 1). As for the T1S1 retrocopies in Carnivora, only partial E1 was identified while the adjacent downstream elements including E302, E3, 3′ TSD and the homologous downstream flanking region were all missed. Since the synteny of flanking protein-coding genes supports the common origin of this insertion site, it is likely that a Carnivora-specific long deletion had occurred after the retrotransposition event.

The T1S2 retrocopies were identified in all species of Laurasiatheria except hedgehogs, which implies that the T1S2 event arose in the common ancestor of Laurasiatheria (Fig. 2). The absence of T1S2 retrocopy in hedgehogs might also be attributed to a lineage-specific deletion of this genomic region. Interestingly, we found that the homologous T1S2 TSD sequences in other clades outside the Laurasiatheria (i.e., Euarchontoglires, Afrotheria, and Xenarthra) are interrupted by a ~ 130 bp fragment. This finding suggests that the effective insertion site probably was not presented in the ancestral genome. The T1S2 retrotransposition event should emerge after the formation of the insertion site generated by the loss of this ~ 130 bp fragment in Laurasiatheria (Fig. 1).

The T1S3 retrocopies were found in most species of Laurasiatheria except bats, hedgehogs and moles (Fig. 2). It is possible that the T1S3 insertion event occurred in the common ancestor of Laurasiatheria or after the divergence of hedgehogs, moles, and other species. In contrast, the T1S4 insertion is bat-specific and contains a deletion in the 3′ end of E302 and a 5′ shortened E1 by 12 bp in length compared to other retrocopies (Fig. 1), which implies the existence of a possible alternative transcription start site (TSS).

### The MIR302CHG retrocopies without the mir-302a homologous regions

For the retrocopies without exon E302, as indicated in Fig. 2, eight independent events corresponding to eight insertion sites (T2S1 ~ T2S8) were identified. The insertions of T2S1 and T2S4 retrocopies occurred in the ancestor of Carnivora. T2S4 retrocopies have a three-nucleotide extension at the 5′ end of E1 (Supplementary MSA file). It is unclear whether this longer E1 resulted from accumulated mutations after retrotransposition or from another alternative TSS. The event of T2S2 insertion happened in either the common ancestor of Ruminantia (cattle) and Cetacea (dolphins and whales) or the common ancestor of Cetartiodactyla and got lost in pigs. The events of T2S3 and T2S5 are lineage-specific in horses and humans, respectively. A possible L1-mediated deletion^44^ was also recognized in T2S5. These five kinds of retrocopies mentioned above were derived from the splice site SS2, while the other three retrocopies described below were from another splice site SS1 (Fig. 1). The T2S6 retrocopy specifically found in flying lemurs shows a 5′ extended E1 by ~ 44 bp in length, which also implies a possible alternative TSS (Fig. S4). The remaining ones, T2S7 and T2S8, are bat-specific retrocopies.

### The alternative splice sites of the *MIR302CHG* gene

For most MIR302CHG retrotransposition events (T1S1 ~ T1S4 and T2S1 ~ T2S5), their parental genes used SS2 as the splice site (Fig. 2). We also noticed that the sequences of SS2 among most current mammalian *MIR302CHG* genes (including marsupials and platypus) are highly conserved and seem to remain functional nowadays. This coherence of SS2 usage between the past retrotransposition events and the current *MIR302CHG* genes suggests that SS2 might be the splice site mainly used in most cases.

However, we also found that bats have one specific retrocopy derived from SS2 site (T1S4), and two specific retrocopies from SS1 site (T2S7 and T2S8) (Fig. 2). Although the SS1 site seems to be mutated (deleted) in the current bat *MIR302CHG* gene (Fig. S4), the three young retrocopies mentioned above suggest that both SS1 and SS2 sites were functional and probably were alternatively spliced in the bat ancestors. It could be noticed that the sequence of SS1 is also conserved in many current mammalian *MIR302CHG* genes (Fig. S4). This fact suggests a possibility that the alternative splicing pattern of SS1 and SS2 may exist not only in bats, but also in other mammalian species.

In the current human *MIR302CHG* gene, the SS2 splice site is mutated and should be functionless (Fig. S4). Therefore, SS1 is supposed to be obligatorily used. Considering that most of the identified retrotransposition events used SS2 as the splice site in the past, it is likely that the ancestral human genome had switched from using two alternative splice sites to fixation in only SS1. This might explain why SS1 is the site annotated in NCBI for the current human *MIR302CHG* gene, while SS2 seems to be the mainly used splice site in most other cases. This switch of splicing patterns might have also occurred independently in treeshrews and the Glires.

### The retrocopies derived from mir-373 and mir-498 in primates

In addition to MIR302CHG retrocopies, we found more microRNA retrotransposition cases derived from the other two parental genes, mir-373 and mir-498. The former generated two independent marmoset-specific retrotransposition events (named 373-S1 ~ S2), while the latter generated four independent primate-specific retrotransposition events (named 498-S1 ~ S4) (Fig. 2). The 498-S1 insertion occurred in the Haplorhini, the common ancestor of Simiiformes (apes, Old World monkeys, and New World monkeys) and tarsiers. The 498-S3 insertion emerged before the divergence of aye-ayes and the lineage containing mouse lemurs (M_Lemur) and sifakas. The 498-S2 and 498-S4 retrocopies are lineage-specific in tarsiers and flying lemurs (F_Lemur), respectively. Unlike the MIR302CHG retrocopies, where the mir-302a homologous region comes from only a part of the middle exon (E302), the entire mir-373 and mir-498 retrocopies are homologous to the currently annotated mir-373 and mir-498 precursors, respectively (Fig. 1 and Supplementary MSA files). All these retrocopies contain the hallmarks of L1-mediated retrotransposition described in the previous sections, which suggests their possible origins. We did not find any potential core promoter elements, nor evidence to support their expression (Table S3) and sequence conservations (Table S4). Therefore, we also consider mir-373 and mir-498 retrocopies as pseudogenes.

## Discussion

### The identified retrocopies emerged from a cellular environment with especially high expression of retrotransposons

In this study, we provide the first cases of microRNA related retrocopies, which were generated via the same L1-mediated retrotransposition machinery as protein-coding genes. Their parental genes include mir-302a and mir-373 that are predominantly expressed in the ESC^30,34–37^, and mir-498 that is predominantly expressed in the placenta^38–40^. It was previously reported that L1-mediated retrotransposition frequently occurred at the early human embryonic developmental stage^45,46^. In addition, comparing with the embryo, L1 might be even more highly activated in the placenta due to its relatively hypomethylated environment and accordingly the reduced repression of transposable elements^47,48^. The elevated retrotransposon activity may thus simultaneously promote the retrotransposition of the also highly expressed mir-302a, mir-373 and mir-498 in the embryonic cells and the placenta. This speculation provides a possible explanation for why our first microRNA retrocopy findings were generated from these three tissue-specific microRNA genes.

### Further evidence for the alternatively spliced exon E302 of the *MIR302CHG* gene

The newly recognized exon E302 was never reported previously. In contrast, the currently annotated exon E2 in the human transcript NR_146093 is missed in all our identified retrocopies. Barroso-delJesus et al. had studied the overall similarity of the *MIR302CHG* gene in humans, mice, dogs, and cattle^35^. According to their similarity plot, we noticed that the interval regions between adjacent mir-302b and mir-302c, and between adjacent mir-302d and mir-367 (referring to Fig. 1 for genomic coordinates), are considerably conserved among species. However, it was extremely diverged at the two flanking intervals of mir-302a (i.e., the region between mir-302c and mir-302a, and the region between mir-302a and mir-302d), suggesting their divergent selection forces. Notably, the sharp borders splitting the conserved and divergent regions in the similarity plot were coincidently in line with the E302 boundaries proposed in this study. Considering the fact that exons usually tend to be much more conserved than introns, these borders also support the existence of exon E302 and its adjacent intron–exon junctions. In summary, the E302 boundaries inferred from the retrocopies were confirmed by both the expression evidence (the NGS reads that span either E1-E302 junction or E302-E3 junction) and the conservation evidence. Hence, the newly identified retrocopies help to reveal the novel scheme of alternative splicing and the ancestral transcripts of the parental gene *MIR302CHG*.

### The possible origins of mir-373 and mir-498 retrocopies

It should be noted that the entire mir-373 and mir-498 retrocopies are homologous to the currently annotated mir-373 and mir-498 precursors, respectively (Fig. 1); which means, the poly-A tails were directly attached to the 3′ end of their parental microRNA precursors. The transcriptional mechanism of mir-371–373 had been well characterized^42^. However, both the upstream conserved promoter that was validated in mice and the downstream canonical polyadenylation signals (PASs) found in human mir-371–373 suggest that the primary transcripts of mir-371–373 clusters should be much longer than the mir-373 precursor in both 5′ and 3′ ends. In other words, the primary transcripts could not serve as the parental source for mir-373 retrocopies directly. It is likely that the 5′ end of the parental sources were generated by Drosha cleavage. However, how the poly-A tails were attached to the 3′ end of the parental sources and not cleaved by Drosha is still unclear. Some yet unknown mechanisms might be involved in the process.

Different from the mir-371–373 cluster, the transcriptional mechanism of mir-498 in C19MC is still under debate. A possibility had been previously proposed that the transcription of C19MC members might be initiated from the internal polymerase III promoter harbored in the tandemly repeated Alus^43^. However, no proximal sense Alu element was identified in the upstream region of mir-498. In contrast, some other studies suggested that C19MC members could be expressed as intronic byproducts when the conserved and tandemly repeated exons were alternatively joined^22,38^. Nonetheless, this intronic byproduct model comes into conflict with the fact that retrocopies should be generated from the end element of a transcript with an attached poly-A tail but not from the internal intron. Although the mechanism generating mir-373 retrocopies has not yet been completely resolved, we hypothesize that the same mechanism might likewise generate mir-498 retrocopies. In other words, similar to mir-373, mir-498 might also be the end element of a long primary transcript. Coincidently, different from the other downstream microRNAs, the first three microRNAs in C19MC, mir-512, mir-1323, and mir-498, all lack the sense Alu and the tandemly repeated exon in their upstream regions. This finding implies that these three microRNAs might have a different regulatory mechanism from the other downstream microRNAs in C19MC.

### The possible impacts of microRNA retrocopies

Similar to microRNAs, some pseudogenes have been reported to have the potential to regulate gene expression and thus may play both oncogenic and tumor-suppressive roles in various aspects of tumorigenesis^49,50^. All the microRNA retrocopies identified in this study are regarded as pseudogenes because they have accumulated a number of mutations and without evidence to support their expression. However, it should be noted that some retrocopies might have a chance to be co-expressed as intronic byproducts or be regulated by potential core promoter elements. In case, under a certain circumstance, these microRNA retrocopies were abnormally expressed, the generated antisense transcripts (e.g., from retrocopies locating in the opposite strand of introns) may hybrid with the mRNAs of their parental genes and thus disturb the expression of the genes targeted by their parental microRNAs. On the other hand, the E1 and E3 regions of transcripts derived from MIR302CHG retrocopies (including T1 and T2) may also compete for RNA binding proteins with the mRNAs of their parental genes. All these scenarios may potentially cause abnormal cellular conditions.

However, before our study, no microRNA retrocopies were reported, and hence no such analysis could be carried out. The reason might be attributed to the short lengths of microRNAs, which may reduce the statistical significance of sequence comparison. We used iterative search strategy and sequence profiles to overcome this problem. A comparative analysis of three genome-wide studies for retrocopies derived from protein-coding genes suggested that using insertion site information to identify retrocopies would have better reproducibility than using exon-exon junctions alone^10^. Utilizing the hallmarks of L1-mediated retrotransposition, including flanking TSDs and poly-A tracts to identify novel retrocopies, would be even more reliable^10^. Since all cases identified in our study exhibit such hallmarks, we should provide clear evidence demonstrating that microRNAs can generate retrocopies via L1-mediated retrotransposition as protein-coding genes. More microRNA retrocopies might be undiscovered yet. It is worthy of conducting a global screen of microRNA retrocopies in order to fulfill the annotation, which may also contribute to the studies of gene expression and regulatory mechanisms.

## Materials and methods

### Genome sequences

The whole-genome assemblies of 45 mammalian species were recruited, including 40 Eutheria species, 4 Marsupials, and the platypus. Their genome assemblies were fetched from the NCBI Assembly database if the RefSeq assemblies are available (accession prefix in GCF). For those species without RefSeq assemblies, their GenBank assemblies (accession prefix in GCA) were used instead. See Table S1 for accession numbers and versions.

### Identification of microRNA retrocopies

Some of our investigated species have their mir-302 stem-loop sequences available in miRBase version 22 (https://www.mirbase.org/). These stem-loop sequences were collected and used as the start cohort to build HMM profiles. The four subtypes (mir-302b, mir-302c, mir-302a, and mir-302d) were treated separately. The constructed profiles were subsequently used for whole-genome scan by the nhmmer program of HMMER 3.2.1 (https://hmmer.org) under default parameters and cutoff. For each iterative scanning process, the profiles were renewed by manual realignment with the newly identified hits. By doing so, the possible targets in more divergent species could have the chance to be identified when their closely related species were included in the profile. The synteny information was utilized to determine their genomic coordinates. Eventually, all newly identified hits located outside the current mir-302–367 cluster region were regarded as retrocopies. We used the same procedures to identity the homologous regions for E1 and E3 exons in *MIR302CHG* and for mir-373 and mir-498 stem-loop sequences.

### Identification of orthologous flanking regions in the species without the certain retrocopy

For each retrocopy, the flanking sequences from both ends were joined into a single fragment. The fragments derived from the same retrotransposition event were thus collected to build an HMM profile. This profile was subsequently used to iteratively scan all other species without the certain retrotransposition event by the procedures described in the previous section. The chosen lengths of these HMM profiles varied from 100 to 400 bp, depending on whether the required specificity could be achieved or not. Ideally, the identified hits should locate in the syntenic region. However, in case no hit was obtained in this area, we would rescan only this syntenic region instead of the whole genome to reduce background noise. If, unfortunately, still no hit obtained, we would go back to verify the results obtained from the whole genome scanning and select the best hit as our candidate fragment. This non-syntenic fragment might encounter genomic rearrangements.

### Multiple sequence alignment

Multiple sequence alignment (MSA) was generated using MEGA 7^51^ with manual gap adjustments. The lineage-specific repeats identified by RepeatMasker (A.F.A. Smit, R. Hubley and P. Green, RepeatMasker Web Server 4.0.8, https://www.repeatmasker.org) or some other species-specific short insertions were manually removed to obtain a clean sequence.

### Secondary structure prediction

For each recognized T1 retrocopy, the certain fragment homologous to human mir-302a stem-loop region was identified and used to infer its own possible stem-loop sequence and perform the secondary structure prediction. As for mir-373 and mir-498 retrocopies, the sequence of the entire retrocopy was used. The secondary structure prediction was achieved by calculating its minimum free energy (MFE) using ViennaRNA Package 2.0^52^ on RNAfold Web Server with default parameters. In some cases, the possible stem-loop sequences might be disrupted by insertions of transposable elements or accumulated simple repeats. To reveal their probable original secondary structures before the disruption, we removed these repetitive elements and performed the structure prediction once again.

### Verification of transcripts containing E302

Numerous NGS runs (prefix in SRR) were selected from NCBI SRA database using “human embryonic stem cell” as the keyword. The E1, E302 and E3 regions of human *MIR302CHG* gene were joined to serve as the query sequence. We used online BLAST to search this query sequence against the above NGS runs one by one, and attempted to look for any paired-end reads that span the junction between E1 and E302, or the junction between E302 and E3.

### MicroRNA expression profiles derived from NGS datasets

To clarify whether the identified retrocopies were expressed, four selected miR-Seq datasets were downloaded from the NCBI SRA database (Table S3). These four studies used NGS platforms to sequence size-selected fragments, which might belong to microRNA mature sequences, obtained from embryonic stem cells or placental cells. The fetched reads in a minimum length of 15 bp after adapter trimming were kept and mapped onto the target sequences using bowtie^53^ with perfect matches. The target sequences included microRNA stem-loop regions from the parental genes and the homologous regions from retrocopies. A target sequence was regarded as expressed if mapped by at least one read.

### Conservation estimation of the microRNA homologous regions in retrocopies

The species tree of the 45 mammalian species (Fig. 2) was adopted from Churakov et al.^54^ and Meredith et al.^55^. Based on this species tree and the current mir-302a stem-loop sequences from all the 45 species, the ancestral sequence for each internal node could be inferred utilizing the principle of Maximum Parsimony by MEGA7^51^. The orthologous regions for all the species were speculated based on the stem-loop sequence of human mir-302a annotated in miRBase. The ambiguous site in the ancestral sequence was designated as ‘N’. Thus, the possible ancestral stem-loop sequence for each T1 retrotransposition event could be obtained and used to compare with the mir-302a homologous regions of all the generated T1 retrocopies. The same procedures were applied for mir-373 and mir-498 retrocopies in the primate lineage, except for their homologous sequences used for calculation were inferred from the retrocopies because the lengths of the retrocopies are shorter than the annotated stem-loop regions. The substitution number between each retrocopy and the possible ancestral sequence was accordingly calculated. The possible time period of occurrence of each retrotransposition event (Fig. 2) was obtained from TimeTree database^56^: 105 ~ 96 Mya for T1S1, 96 ~ 89 Mya for T1S2, 89 ~ 78 Mya for T1S3, 79 Mya ~ present for T1S4, 74 ~ 67 Mya for 498-S1, 67 Mya ~ present for 498-S2, 59 ~ 55 Mya for 498-S3, 76 Mya ~ present for 498-S4, and 46 Mya ~ present for 373-S1 and 373-S2. Combining these time frames and the average mammalian genome mutation rate, 2.2 × 10^–9^ per base pair per year^57^, we could estimate the frequency of expected substitutions that arose since the occurrence of each retrotransposition event, and transform it into expected observed substitution frequency using JC69 one-parameter model^58^. Consequently, for each retrocopy, the expected observed substitution frequency could be used to calculate the binomial probability of finding the actual observed substitution number (or less) between the certain retrocopy and its possible ancestral sequence under the null hypothesis that the substitutions were neutrally accumulated (Table S4).

## Supplementary information

Supplementary Figures.

Supplementary Tables.

Supplementary MSA File 1.

Supplementary MSA File 2.

Supplementary MSA File 3.
