# Supplementary material for: MicroRNA retrocopies generated via L1-mediated retrotransposition in placental mammals help to reveal how their parental genes were transcribed
Source: Sci Rep. 2020 Nov 26;10:20612. doi: 10.1038/s41598-020-77381-8 (PMC7692494; doi:10.1038/s41598-020-77381-8)
Supplement: Supplementary file 1 — Supplementary Figures. [file 41598_2020_77381_MOESM1_ESM.pdf]

Supplementary Materials

MicroRNA retrocopies generated via L1-mediated retrotransposition in placental mammals help to reveal how their parental genes were transcribed

Cheng-Tsung Pan[1], Yeong-Shin Lin[1,2,3,*]

[1] Institute of Bioinformatics and Systems Biology, National Chiao Tung University, Hsinchu 300, Taiwan

[2] Department of Biological Science and Technology, College of Biological Science and Technology, National Chiao Tung University, Hsinchu 300, Taiwan

[3] Center For Intelligent Drug Systems and Smart Bio-devices (IDS^{2}B), National Chiao Tung University, Hsinchu 300, Taiwan

* Author for Correspondence: Yeong-Shin Lin, Institute of Bioinformatics and Systems Biology, National Chiao Tung University, Hsinchu 300, Taiwan, yslin@faculty.nctu.edu.tw

Supplementary figures

Fig. S1.

Fig. S2.

Fig. S3.

Fig. S4.

Fig. S5.

Fig. S6.

Description of supplementary MSA files

Supplementary Excel file

Table S1. Genome assemblies used for 45 mammalian species in this study.

Table S2. The genomic information for each identified retrocopy and the independent transposable element insertion events.

Table S3. The microRNA expression validation results.

Table S4. Conservation estimation of the microRNA precursor homologous regions in retrocopies.

Fig. S1. The typical hallmarks of L1-mediated retrotransposition recognized in (A) MIR302CHG retrocopies, (B) mir-373 retrocopies and (C) mir-498 retrocopies. This figure is composed of screenshots cropped from the three supplementary MSA files. The black rectangles indicate the identified TSDs for each retrotransposition event except for T2S5, which was accompanied by a possible L1-mediated deletion without the generation of TSDs. The character “D” in MSA represents removed insertions, simple repeats, or omitted fragments without specifying their actual sequence lengths. The character “B” in MSA separates two discontinuous regions resulted from genomic rearrangements. Boundaries of the inserted retrocopies are denoted by the characters “SS.”

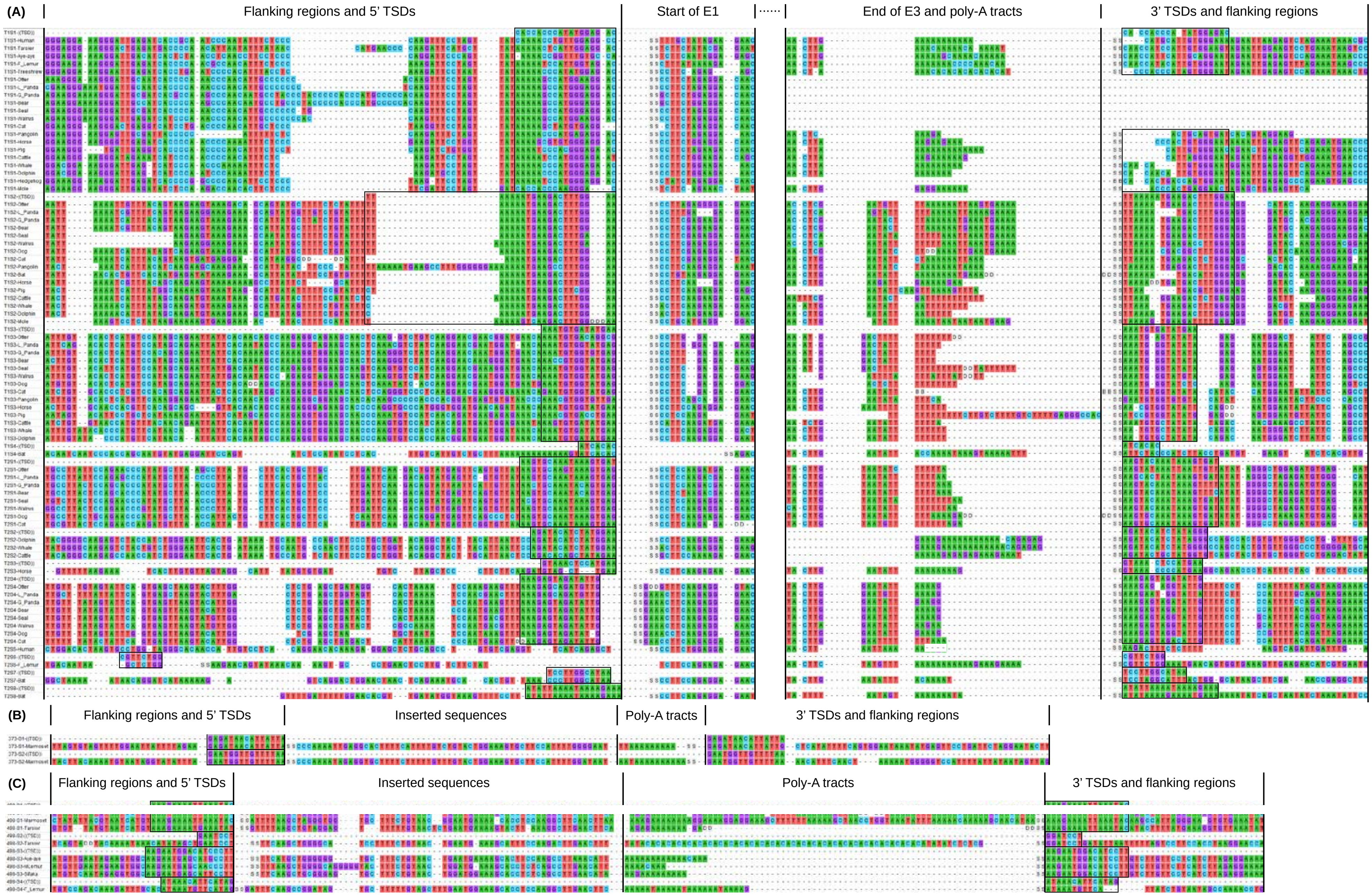

Fig. S2. The sequence comparison of exon E302 and its flanking splice sites between the parental *MIR302CHG* genes (PG) and the derived retrocopies among various mammalian species. The exon-exon junctions in both two types of retrocopies are in line with the splice sites in their parental genes, supporting the existence of the newly identified exon E302. The black and red triangles and bold lines indicate the junctions of previously annotated and newly proposed alternative splice sites, respectively. The two alternative splice sites SS1 and SS2 located at the 3' end of exon E1 described in **Fig. 1** are also specified. The nucleotides with a green background represent the canonical GT-AG intronic splice sites.

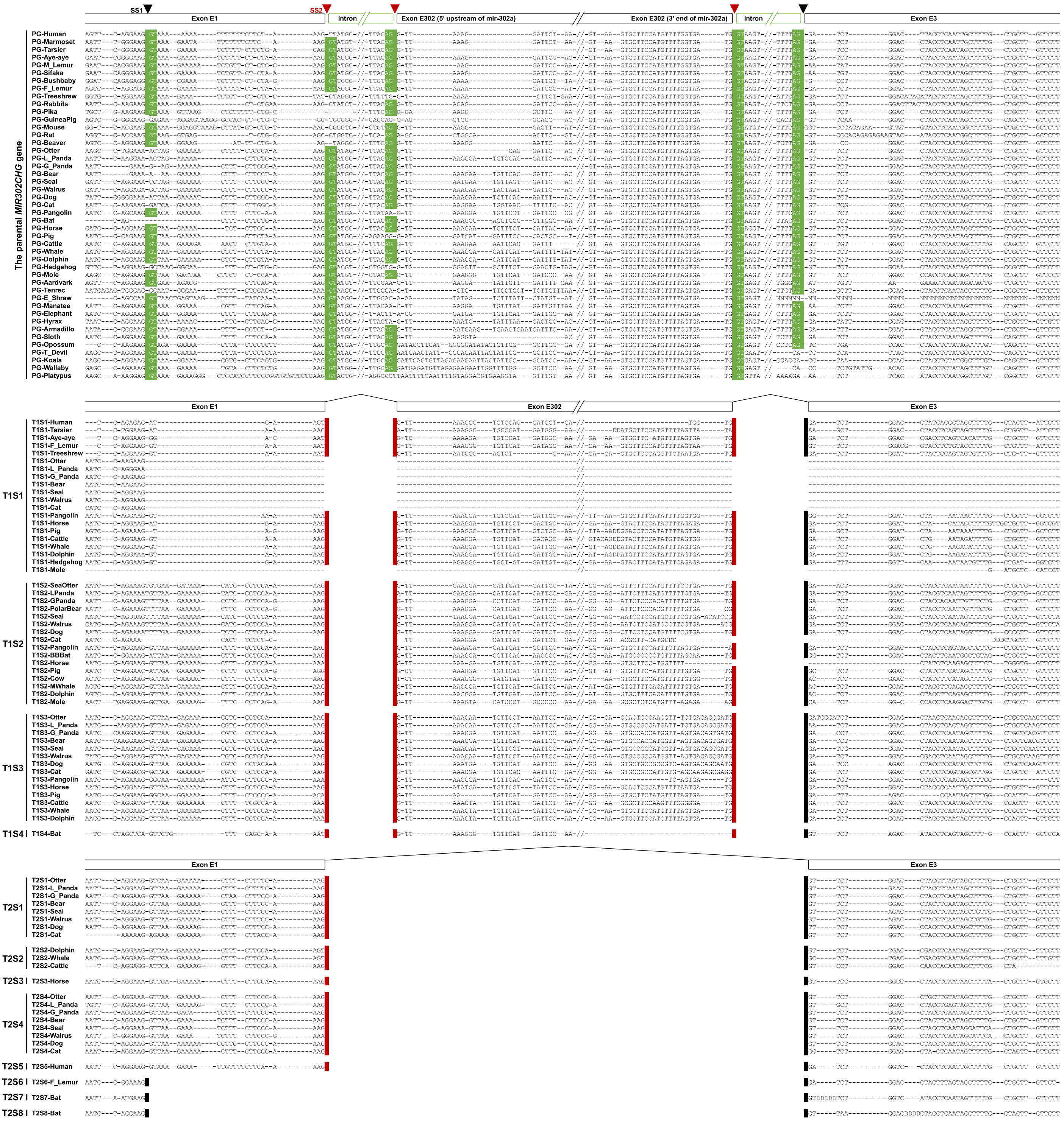

Fig. S3. The transcripts containing exon E302 verified by NGS paired-end reads that spans the junction between E1 and E302 or the junction between E302 and E3. Six paired-end reads from four runs (representing four samples) of three independent studies were selected and displayed as the examples. All these reads are perfectly aligned to the parental E1, E302 and E3 regions.

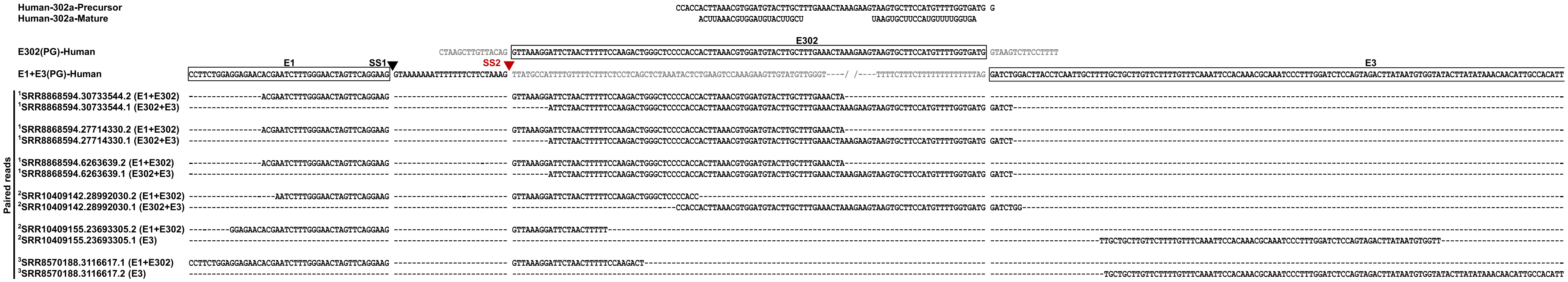

[1] Human embryonic stem cell from SRA study SRP191503

[2] Human embryonic stem cell H9 (SRR10409142) and induced pluripotent stem cell XFiPSC (SRR10409155) from SRA study SRP228826

[3] Human embryonic stem cell CH2 from SRA study SRP185781

* The adapter regions were trimmed and not shown in this figure

Fig. S4. The sequence comparison of exon E1 and its flanking regions between the parental *MIR302CHG* genes (PG) and four derived retrocopies among various mammalian species. The transcription start site (TSS, blue bold lines with arrows) and the splice site SS1 (the black inverted triangle and black bold lines) were validated and annotated in the current human *MIR302CHG* gene. In contrast, two alternative transcription start sites were inferred from the retrocopies T1S4 and T2S6 (yellow bold lines with arrows) in this study. Similarly, the alternative splice site SS2 (the red inverted triangle and the red bold line) was also inferred from most our identified retrocopies. Except for the displayed four retrocopies, all the other omitted retrocopies were derived from the splice site SS2 and the general annotated TSS. The nucleotides with a green background indicate the canonical intronic splice sites. We can notice that SS1 and SS2 may potentially co-exist (both with a green background) in many mammalian species at present. In contrast, in humans, treeshrews and Glires, their SS2 splice sites are currently mutated and disabled. Site SS1 is supposed to be obligatorily used for them.

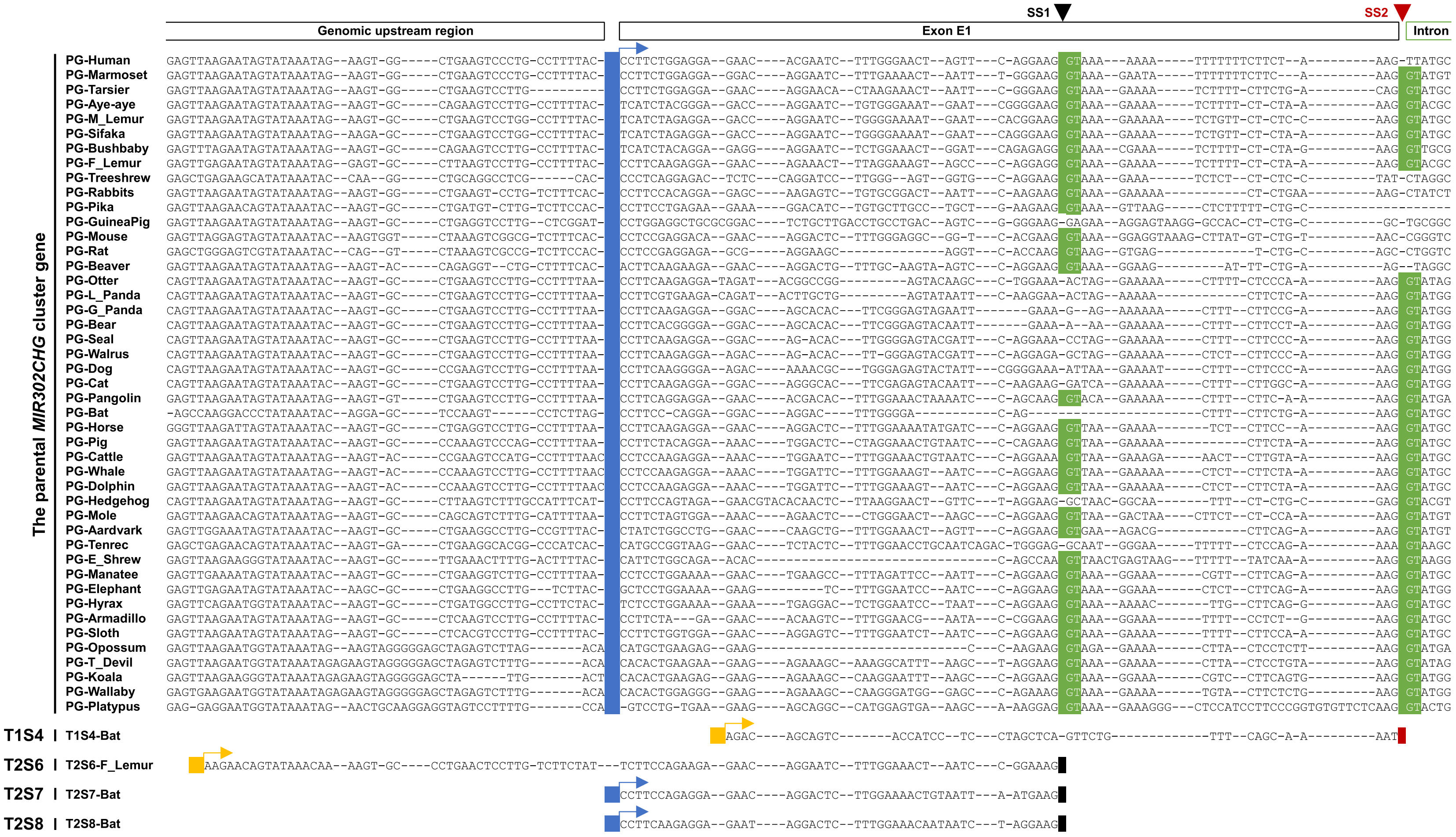

Fig. S5. Possible core promoter elements found in the exon E1 and upstream regions of T1S1, T1S2 and T1S3 retrocopies. Species were colored accordingly to their superorders: Euarchontoglires (blue), Laurasiatheria (green), Afrotheria (red), and Xenarthra (orange). The TATA-box and mammalian initiator patterns were referred from Smale and Kadonaga[1], and highlighted on the alignment with orange and purple backgrounds, respectively.

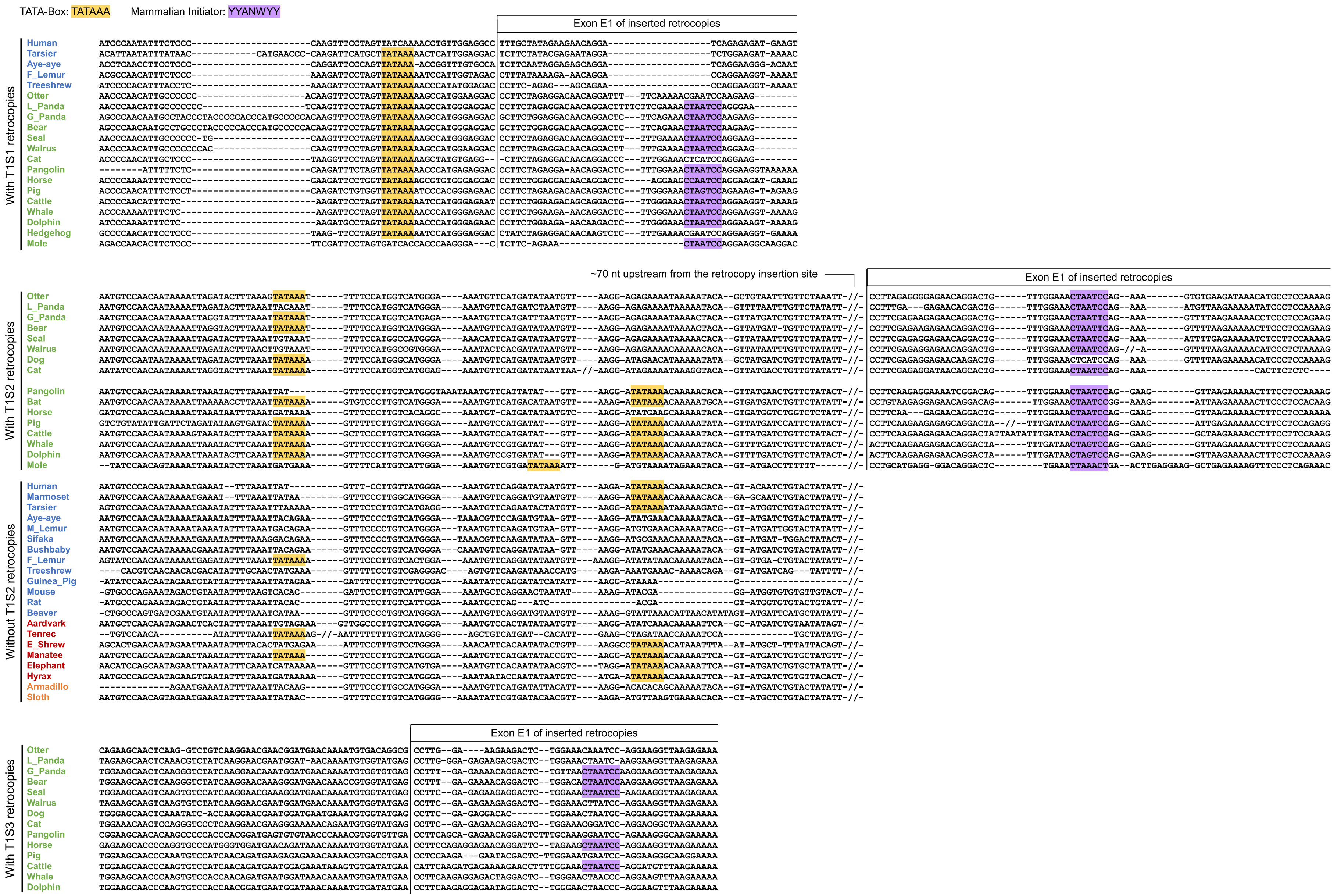

Fig. S6. The predicted secondary structures of the mir-302a precursor homologous regions of (A) T1S1, (B) T1S2, (C) T1S3 and the bat-specific T1S4 retrocopies, (D) the entire mir-373 retrocopy and (E) the entire mir-498 retrocopy by RNAfold Web Server. In some cases, the inspected sequences were probably disrupted by insertions of transposable elements (TEs) or accumulated simple repeats. If their hairpin structures could be regenerated after removing these repetitive elements, the re-evaluation results would be represented instead and denoted by an asterisk (*). The "(rc)" tag indicates that the prediction and evaluation results were generated from the reverse complement sequence.

(A) T1S1

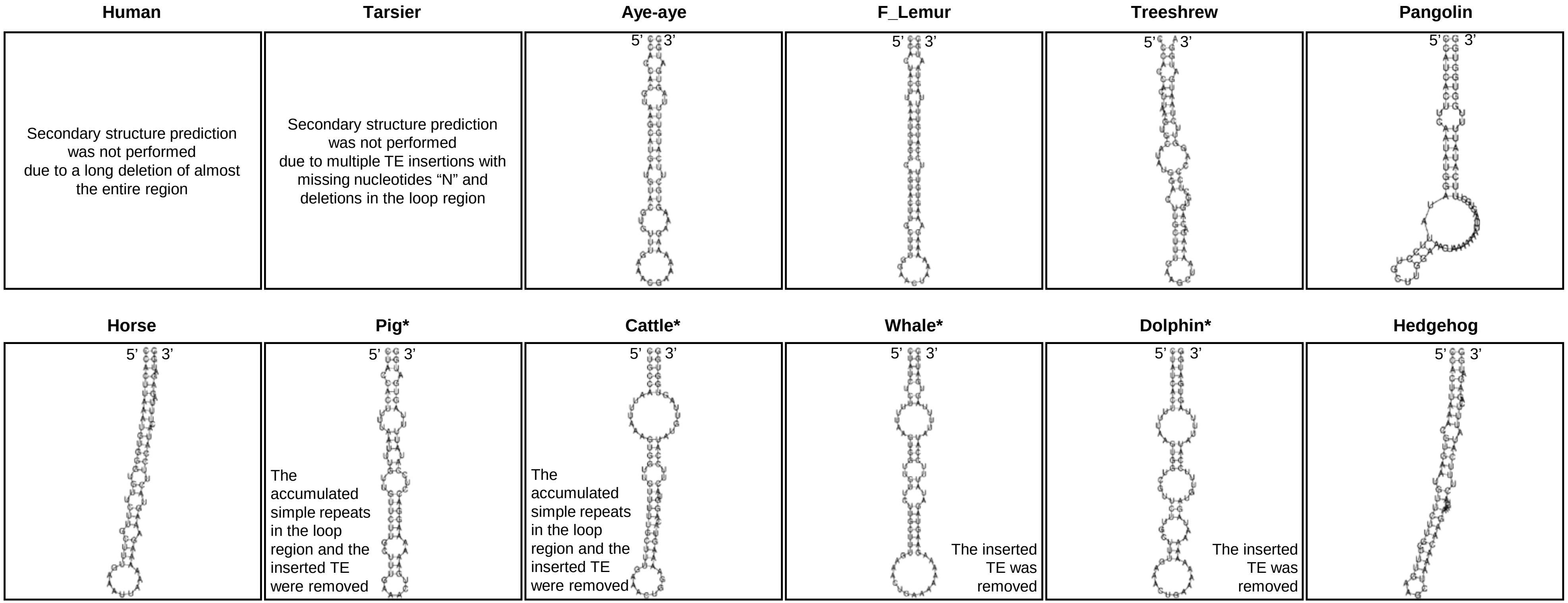

(B) T1S2

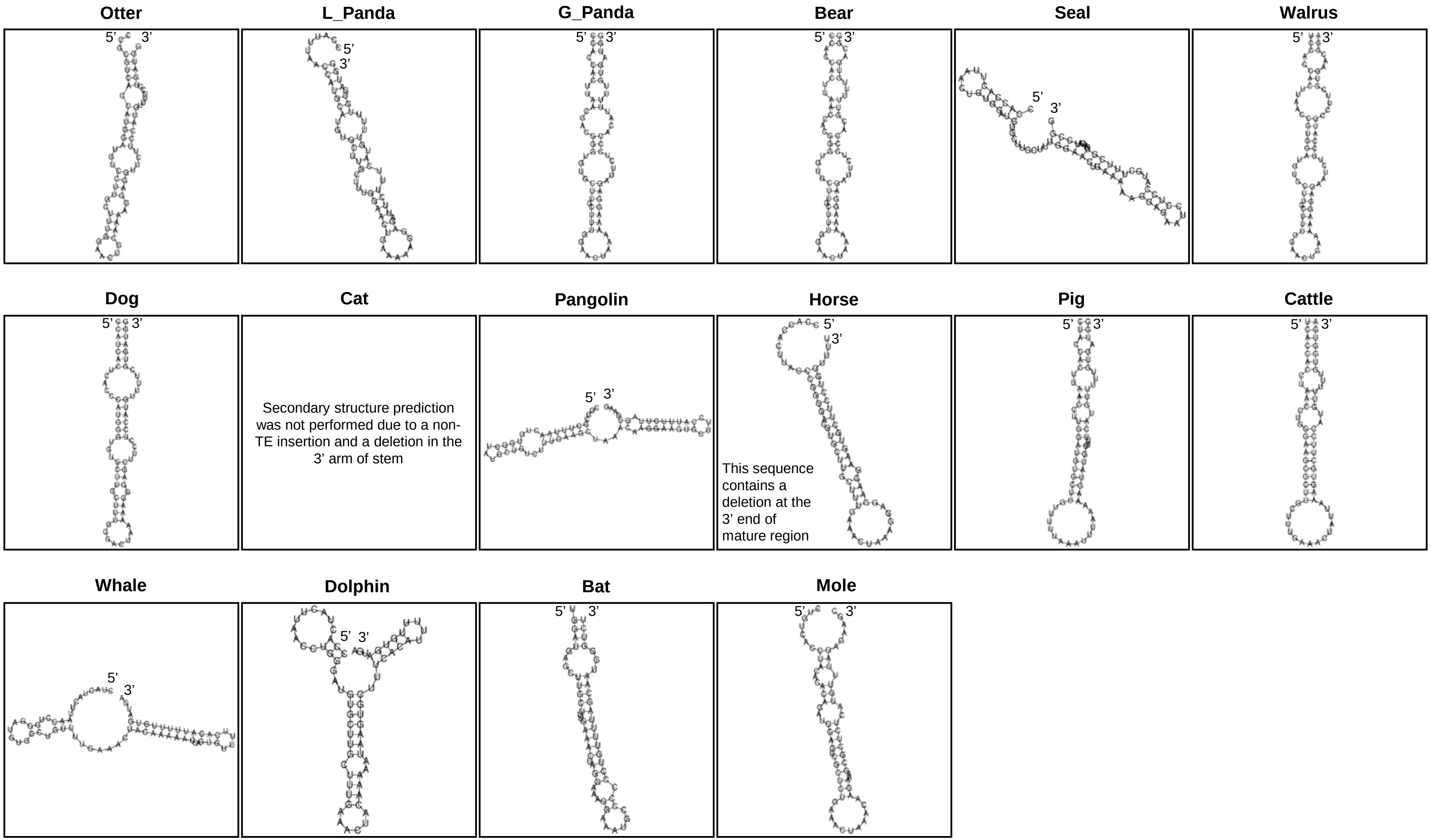
Otter
L_Panda
G_Panda
Bear
Seal
Walrus
Dog
Cat
Secondary structure prediction was not performed due to a non-TE insertion and a deletion in the 3’ arm of stem
Pangolin
Horse
This sequence contains a deletion at the 3’ end of mature region
Pig
Cattle
Whale
Dolphin
Bat
Mole
5’
3’

(C) T1S3 and bat-specific T1S4

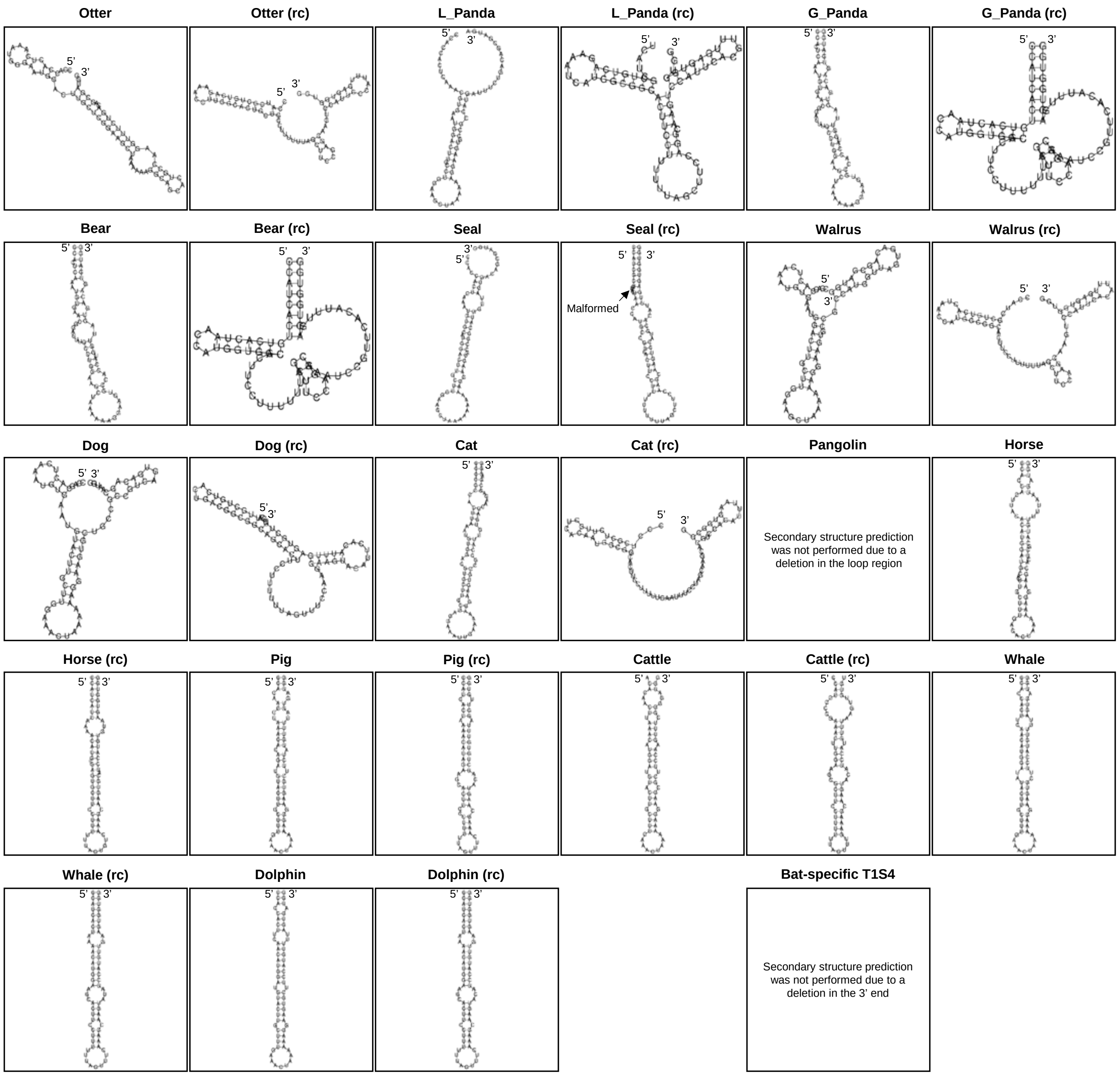

(D)

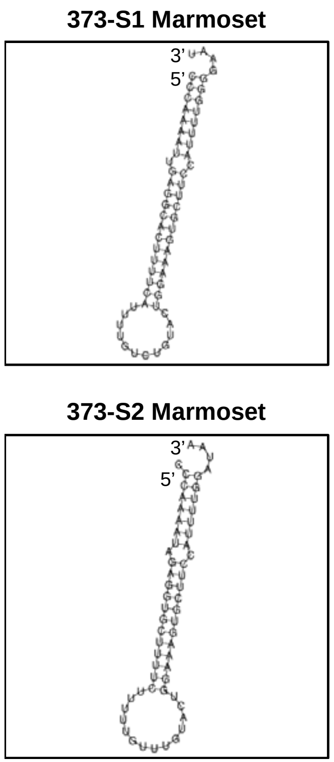
373-S1 Marmoset
373-S2 Marmoset

(E)

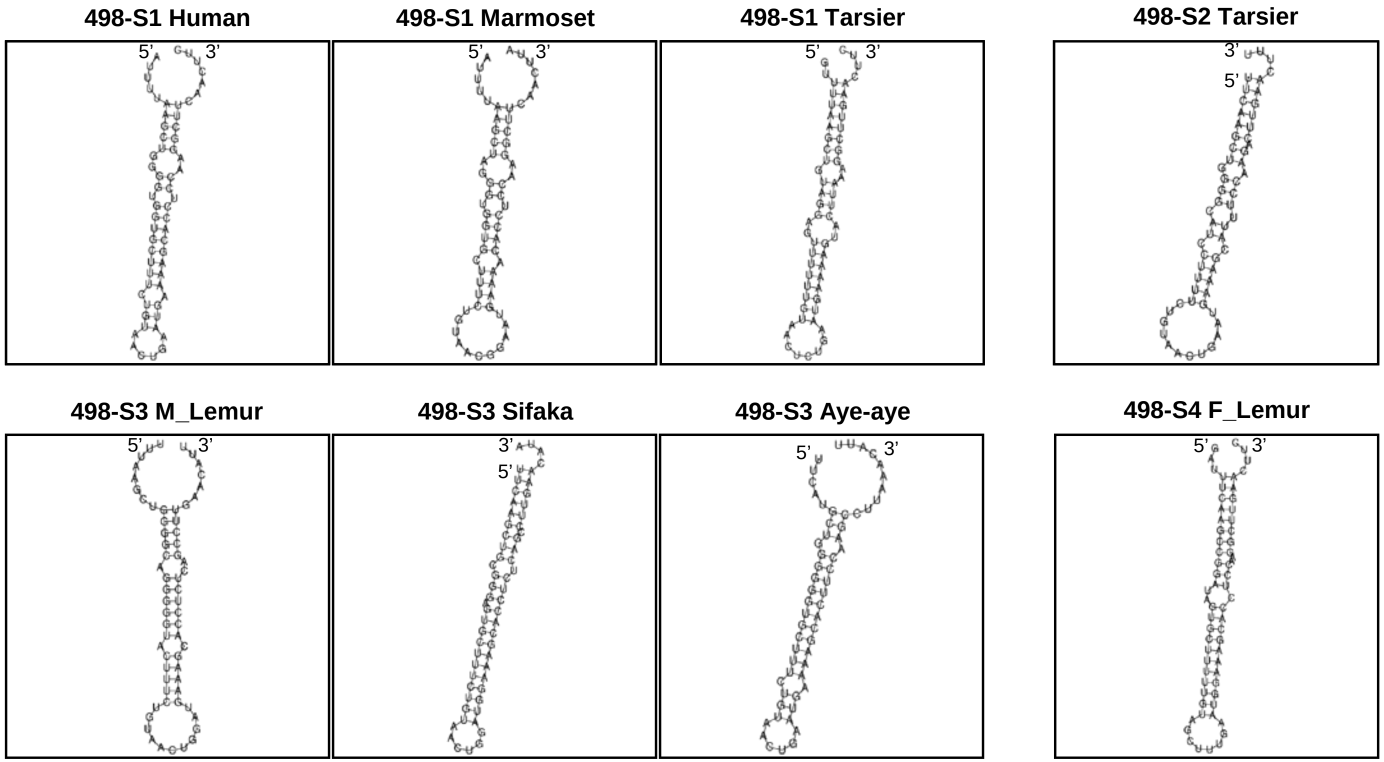
498-S1 Human
498-S1 Marmoset
498-S1 Tarsier
498-S2 Tarsier
498-S3 M_Lemur
498-S3 Sifaka
498-S3 Aye-aye
498-S4 F_Lemur

Supplementary MSA files

Three multiple sequence alignment (MSA) files in FASTA format for MIR302CHG retrocopies, mir-373 retrocopies and mir-498 retrocopies are provided. The sequences of inserted retrocopies were aligned with the corresponding regions in their parental genes. The flanking regions for each retrocopy were also displayed and aligned with the homologous regions in species without the certain retrotransposition event. The identified TSDs for each retrotransposition event are shown in an independent row. The character “D” in MSA represents removed insertions, simple repeats, or omitted fragments without specifying their actual sequence lengths. The character “B” in MSA separates two discontinuous regions resulted from genomic rearrangements within a single scaffold (e.g., T1S1 in hedgehogs) or between different scaffolds (e.g., T1S3 in cats). The type of retrocopies (T1S1~T2S8, 373-S1~S2 and 498-S1~S4), and sequences obtained from the parental genes (PG), DNA transposon insertions (DI), independent SINE insertions (ISI), and species without any insertions (WO) were specified. Boundaries of the inserted retrocopies, the other independent transposable elements inserted at or near our investigated retrotransposition sites, and the parental mir-373 and mir-498 microRNA precursors, or the transcription start sites (TSS) of the parental *MIR302CHG* genes are denoted by the characters “SS.” The single character “S” indicates the splice junctions. Note that the aardvark E3 is located within an unassembled region (“N”) in the scaffold, which was inferred according to the conserved downstream sequences (the region outside the MSA).

Reference

1 Smale, S. T. & Kadonaga, J. T. The RNA polymerase II core promoter. *Annu Rev Biochem* **72**, 449-479, doi:10.1146/annurev.biochem.72.121801.161520 (2003).
